# Midnight salivary cortisol secretion and the use of antidepressants were associated with abdominal obesity in women with type 1 diabetes: a cross sectional study

**DOI:** 10.1186/s13098-019-0481-3

**Published:** 2019-10-30

**Authors:** Eva Olga Melin, Magnus Hillman, Maria Thunander, Mona Landin-Olsson

**Affiliations:** 1 Department of Clinical Sciences Lund, Diabetes Research Laboratory, Lund University, Lund, Sweden; 2 Department of Research and Development, Region Kronoberg, Box 1223, 351 12 Växjö, Sweden; 3 Primary Care, Region Kronoberg, Växjö, Sweden; 4 Department of Internal Medicine, Endocrinology and Diabetes, Central Hospital, Växjö, Sweden; 5 Department of Endocrinology, Skane University Hospital, Lund, Sweden

**Keywords:** Abdominal obesity, Alexithymia, Antidepressants, Cortisol, Depression, Physical inactivity, Sex differences, Smoking, Type 1 diabetes

## Abstract

**Background:**

Abdominal obesity is a risk factor for cardiovascular disease. The aim was to explore the influence of midnight salivary cortisol (MSC), antidepressants and sex on abdominal obesity in type 1 diabetes (T1D). We controlled for physical inactivity, smoking, depression and alexithymia.

**Methods:**

Cross sectional study of 190 T1D patients (86 women/104 men, 18–59 years, diabetes duration 1–55 years), consecutively recruited from one specialist diabetes outpatient clinic. Anthropometrics, blood pressure, saliva and blood samples were collected, supplemented with data from electronic medical records. Depression and alexithymia were assessed by self-report instruments. MSC (nmol/l) was categorised into 3 levels: high MSC: (≥ 6.7) (n = 64); intermediate MSC: ≥ 3.7− < 6.7) (n = 64); low MSC (< 3.7) (n = 62). Abdominal obesity was defined as waist circumference (meters) ≥ 0.88 for women and as ≥ 1.02 for men. Multiple logistic regression analyses (Backward: Wald) were performed. The Hosmer and Lemeshow test for goodness-of-fit and Nagelkerke R^2^ were used to evaluate each multiple logistic regression analysis model.

**Results:**

The prevalence of abdominal obesity was three times higher in the women than in the men (24% versus 8%) (*p* = 0.002). Antidepressants were used by 10% of the women and by 4% of the men (*p* = 0.09). The prevalence of high MSC was 1.7 times higher in the women (43% versus 26%); the prevalence of both intermediate MSC (28% versus 38%) and low MSC (29% versus 36%) were lower in the women (*p* = 0.048). Significant associations with abdominal obesity were for all 190 patients: female sex (adjusted odds ratio (AOR) 3.4 (confidence interval (CI) 1.4–8.2)) and the use of antidepressants (AOR 4.3 (CI 1.2–14.8)); for the 86 women: high MSC (AOR 18.4 (CI 1.9–181)) and use of antidepressants (AOR 12.2 (CI 2.0–73.6)); and for the 104 men: alexithymia (AOR 5.2 (CI 1.1–24.9)).

**Conclusions:**

Clear sex differences were demonstrated with a distinct higher prevalence of abdominal obesity, as well as a distinct higher prevalence of high midnight salivary cortisol in the women with type 1 diabetes. High midnight salivary cortisol secretion and the use of antidepressants were independent risk factors for abdominal obesity in the women.

## Background

Type 1 diabetes (T1D) is an autoimmune disease, characterised by insulin deficiency due to pancreatic β-cell loss leading to hyperglycaemia [1]. The incidence of T1D is increasing worldwide, and the incidence is highest in the Scandinavian countries [1]. The overall incidence including both children and adults during the period 1999 to 2001 was 30.3 per 100,000 and year in the County of Kronoberg, Sweden, where this study was performed [2]. The risk is higher among children, up to 40 per 100 000 and year [2]. T1D is associated with increased risk for myocardial infarction, heart failure and ischemic stroke [3]. The risk of death from cardiovascular causes in people with T1D in Sweden is more than twice as high as in the general population [4]. As compared to men, Swedish women with T1D have a significantly greater excess risk of death from cardiovascular disease across all age groups [4].

Abdominal obesity is an important risk factor for cardiovascular disease, and is linked to increased all-cause mortality [5, 6]. Female sex is a risk factor for weight gain and obesity during childhood and adolescence in patients with T1D [7]. Abdominal obesity has been associated with both hyperactivity of the hypothalamic–pituitary–adrenal (HPA) axis and with impaired androgen balance, with increased testosterone levels in women and decreased testosterone levels in men in non-diabetic populations [6]. Exaggerated adrenarche has been observed in children with T1D, leading to hyperandrogenic symptoms in women [8]. There is a strong inter-relationship between stress, activation of the HPA axis, energy homeostasis, and abdominal obesity [6, 9, 10], with higher HPA axis responses to stress in women [6]. Stress and glucocorticoids act to control both food intake and energy expenditure. In particular, glucocorticoids are known to increase the consumption of foods enriched in fat and sugar [9]. Cortisol is metabolised in the adipocytes where 11β-hydroxysteroid dehydrogenase type 1 (11β-HSD1) catalyses regeneration of cortisol from inactive cortisone, and where 11β-HSD2 catalyses conversion of active cortisol to inactive cortisone [6, 10]. High levels of 11β-HSD1 in the adipocytes contribute to abdominal obesity and obesity related disorders such as insulin resistance and T2D [6, 10, 11]. Sex differences have been demonstrated with upregulation of 11β-HSD1 by 17β-estradiol in women and by androgens in men [12].

Several disturbances of the hypothalamus–pituitary–adrenal (HPA) axis in patients with T1D have been demonstrated. The disturbances include increased basal hyperactivity [13], exaggerated nocturnal rise in plasma cortisol [14], greater cortisol responses to corticotropin releasing hormone (CRH) stimulation [15], and impaired glucocorticoid negative feedback [13]. Reduced cortisol inactivation via 5α-reductase has been indicated in children with T1D [8].

Smoking and physical inactivity also have impact on cortisol secretion [16, 17]. Activation of the HPA-axis is linked to atherosclerosis of the carotid and coronary arteries, and cardiovascular morbidity and mortality [6, 18–21].

Antidepressants might contribute to the development of obesity [22]. The use of antidepressants has risen dramatically during the last 30–40 years in parallel with the worldwide epidemic of obesity [22]. According to previous research antidepressants may have effect on the HPA axis, upregulation in the short term and downregulation in the long term [23]. Several antidepressants present pharmacokinetic sex differences accentuated by the gonadal hormones [24]. Depression is commonly accompanied by weight changes, weight loss in melancholic depression and weight gain in atypical depression [25]. We previously found indications that these patients with T1D mainly suffered from symptoms of melancholic depression [26]. Alexithymia is a personality trait increasing the risk for obesity [27]. We previously showed that female sex, alexithymia and physical inactivity were associated with abdominal obesity in these patients [28]. The prevalence of general obesity (BMI ≥ 30 kg/m^2^) in the women with T1D was almost twice that of the women in the Swedish general population, whereas it was not increased in the men with T1D [29]. To further explore potential risk factors for abdominal obesity we now include midnight salivary cortisol (MSC) secretion in the analyses. We hypothesize that MSC secretion and the use of antidepressants contribute to abdominal obesity. The aim was to explore links between sex, MSC, the use of antidepressants and abdominal obesity. We adjusted for physical inactivity, smoking, severe hypoglycaemia episodes, depression and alexithymia.

## Methods

**Fig. 1:**
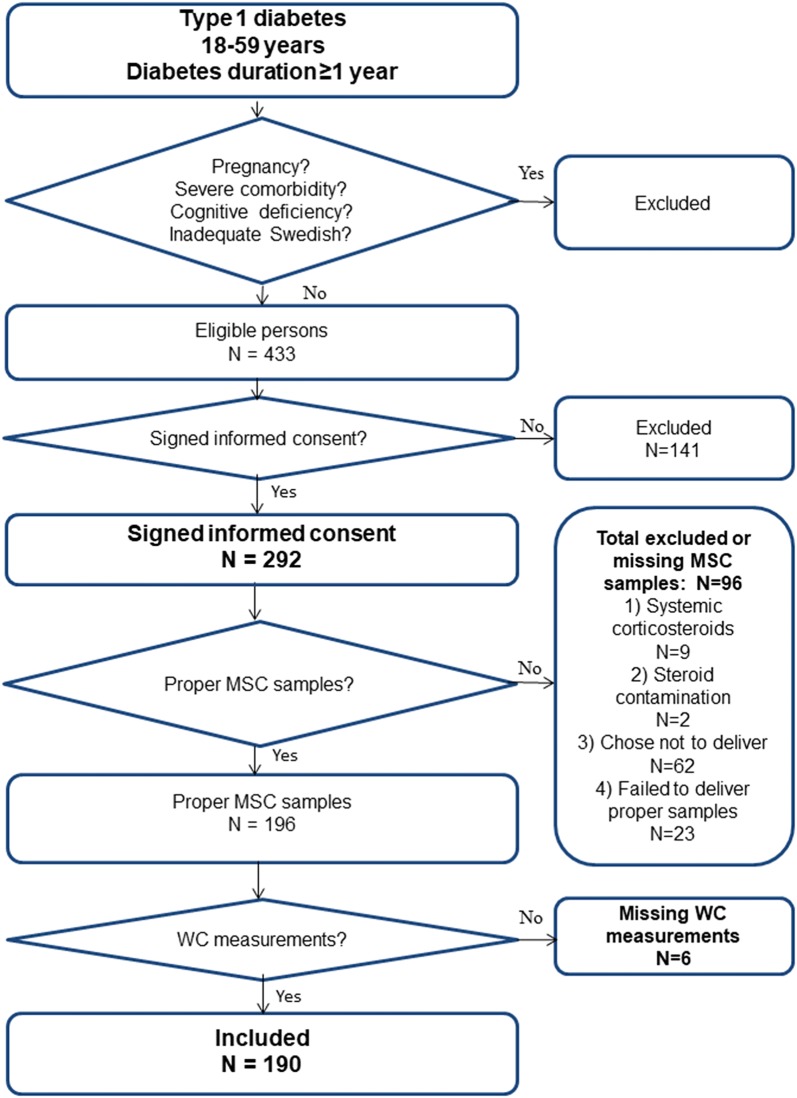
Flow chart for the 190 included participants. Inclusion and exclusion criteria, and missing values are demonstrated

This study of patients with T1D has a cross sectional design. The participants were clinically diagnosed with T1D and were consecutively recruited by specialist diabetes physicians or diabetes nurses at regular follow up visits at one secondary care specialist diabetes clinic in Kronoberg County, Sweden, during the period 03/25/2009 to 12/28/2009. The catchment population was 125,000. Inclusion criteria were T1D, age 18–59 years and diabetes duration ≥ 1 year (see Fig. 1). Exclusion criteria were systemic corticosteroid treatment, pregnancy, severe somatic comorbidities (Cushing’s syndrome, cancer, hepatic failure or end-stage renal disease), severe psychiatric comorbidities (psychotic or bipolar disorder, severe personality disorder, or substance abuse), cognitive deficiency (stroke, mental retardation or dementia), or inadequate knowledge of Swedish. Two hundred and ninety-two eligible patients provided written informed consent to participate. Nine patients using systemic corticosteroids and two patients using topical steroids with very high MSC values (82 and 72 nmol/l) were excluded as steroid contamination was suspected, and 85 patients chose not to deliver or failed to deliver proper samples (Fig. 1) [17]. Proper samples of MSC were delivered by 196 patients, of whom 190 had WC measurements, which rendered 190 included participants (Fig. 1). In our previous studies of abdominal obesity in these patients there were 284 participants [28, 29].

Anthropometrics, blood pressure, saliva and blood samples were collected, supplemented by data from electronic medical records and the Swedish National Diabetes Register (S-NDR) [4, 30]. Depression and alexithymia were assessed by self-report instruments. The MSC measurements were divided into three MSC levels, low, intermediate and high MSC, containing one-third of the MSC samples in each group.

### Anthropometrics and blood pressure

WC, weight, length and blood pressure were measured according to standard procedures by a nurse. Abdominal obesity was defined as WC ≥ 0.88 m for women and as ≥ 1.02 m for men [5]. General obesity was defined as BMI ≥ 30 kg/m^2^ for both sexes [5]. In a backward elimination multiple logistic regression analysis we previously showed that abdominal obesity compared to general obesity had a higher association with HbA1c > 70 mmol/mol [31].

### Midnight salivary cortisol (MSC)

Each patient collected one MSC sample between 23.30 and 00.30 h in their home within 1 week after recruitment, using the Salivette^®^ sampling method (Sarstedt, Nümbrecht, Germany) [17, 26, 32]. Patients had a restriction period of 30 min prior to sampling when they were told not to eat, drink, smoke, use snuff, nor to perform physical exercise, and a period of 60 min prior to sampling when they should avoid brushing their teeth [17, 26]. The samples were centrifuged and frozen at − 25 centigrades until assayed within a year. The Roche Cobas Cortisolassay immunoassay (ECLIA) was used on an Elecsys 2010 immunoanalyser system (Roche Diagnostics, Mannheim, Germany) [17, 26, 33]. The detection limit was 1.9 nmol/l. The intra-assay coefficient of variation was < 3%. Low MSC was defined as < 3.7 nmol/l; intermediate MSC as ≥ 3.7 to < 6.7 nmol/l; and high MSC as ≥ 6.7 nmol/l).

### HbA1c and serum lipids

After an overnight fast, blood samples were collected. HbA1c and serum lipids were analysed with an Olympus AU clinical chemistry analyser with high specificity (Olympus AU^®^, Tokyo, Japan) [34]. The intra-assay coefficients of variation were for HbA1c < 1.2%; total cholesterol < 2.1%; HDL-cholesterol < 3.0%; LDL-cholesterol < 2,6%; and for triglycerides < 2.2%.

### Episodes of hypoglycemia

A severe episode of hypoglycemia was defined as needing help from another person. Episodes during the last 6 months prior to recruitment were registered.

### Smoking and physical inactivity

Smokers were defined as having smoked any amount of tobacco during the last year. Levels of physical activity were assessed by interviews performed by skilled nurses and physicians at the regular follow-up visits. Levels of physical activity performed at work and during leisure time were evaluated. Physical activity, which was defined as at least 30 min of moderate activities, was divided into four levels performed weekly: less than once, 1–2 times, 3–5 times, > 5 times, as registered in the S-NDR [30]. Physical activity was also dichotomized into physical inactivity which was defined as less than 30 min of moderate activities once a week, and physical activity which represents all other levels of physical activity [17, 28, 31].

### Self-report instruments

Depressive symptoms were assessed by the Hospital Anxiety and Depression Scale - the depression subscale (HADS-D), which consists of 7 statements [35]. Each statement has four response alternatives with scores from 0 to 3. The recommended cut-off level was used to define depression as in our previous research: ≥ 8 points [17, 28, 31, 35–38]. A major characteristic of HADS-D is that potential symptoms of somatic disease are not included [35]. Alexithymia was assessed by Toronto Alexithymia Scale-20 items (TAS-20) [27, 39]. It is based on three subscales: “difficulties identifying feelings”, “difficulties describing feelings” and “externally oriented thinking”. TAS-20 consists of 20 statements rated from 1 to 5. The recommended cut-off level was used to define alexithymia as in our previous research: ≥ 61 points [26–28, 31].

### Cardiovascular complications

Cardiovascular complications were defined as ischemic heart disease, cardiac failure, stroke or transient ischemic attacks.

### Medication

The following types of antidepressants were used: selective serotonin reuptake inhibitors (SSRIs) (ATC codes N06AB04 and N06AB10); selective norepinephrine reuptake inhibitors (SNRIs) (ATC code N06AX16); combined serotonin and norepinephrine reuptake inhibitors (ATC code N06AX21); tricyclic antidepressants (ATC code N06AA04); and/or tetracyclic antidepressants (ATC code N06AX11). The use of antidepressants was dichotomized into users and non-users of antidepressants. Lipid lowering drugs (LLD) were defined as equal to HMG CoA-reductase inhibitors (statins) (ATC-codes C10AA). The following antihypertensive drugs (AHD) were used: calcium antagonists (ATC codes C08CA01-02); angiotensin-converting enzyme (ACE) inhibitors (ATC codes C09AA-BA); angiotensin II antagonists (ATC codes C09CA-DA); selective beta-adrenoreceptor antagonists (ATC code C07AB); and/or diuretics (ATC codes C03AA03 and C03CA01).

### Statistical analysis

Analysis of data distribution using histograms revealed that age, diabetes duration, MSC, and WC were not normally distributed. Data were presented as median values (quartile (q)_1_, q_3_; range), and analyses were performed with Mann–Whitney *U* test or Kruskal–Wallis *H* test. Fisher’s exact test or Linear-by-Linear Association (both two-tailed) were used to analyze categorical data. Crude odds ratios (CORs) with abdominal obesity as dependent variable were calculated. Variables with *p*-values ≤ 0.10 for the CORs and age (independent of *p* – value) were entered into multiple logistic regression analyses (Backward: Wald) [40], with abdominal obesity as dependent variable for all, women and men. The Hosmer and Lemeshow test for goodness-of-fit and Nagelkerke R^2^ were used to evaluate each multiple logistic regression analysis model. Confidence intervals (CIs) of 95% were used. *p* < 0.05 was considered statistically significant. SPSS^®^ version 23 (IBM, Chicago, Illinois, USA) was used for all statistical analyses.

## Results

In this study 190 patients with T1D (45% women, age 18–59 years, diabetes duration 1–55 years) were included. The patients used either multiple daily insulin injections (MDII) (90%) or continuous subcutaneous insulin infusion (CSII) (10%). Five women used antidepressants with either the ATC code N06AB04 or N06AB10; one woman used an antidepressant with the ATC code N06AA04; one woman used a combination of antidepressants with the ATC codes N06AB04 and N06AX11; and one woman used a combination of antidepressants with the ATC codes N06AX16 and N06AX11. Three men used antidepressants with the ATC code N06AB04; and one man used antidepressants with the ATC code N06AX21.

Baseline characteristics are presented for all, women and men in Table 1. The prevalence of abdominal obesity was three times higher in the 86 women than in the 104 men (24% versus 8%) (*p* = 0.002).

**Table 1:** Baseline characteristics and comparisons between 86 women and 104 men with T1D

	All patients	Women	Men	*p* ^a^
*N*	190	86	104	
Age (years)	43 (32, 51; 18–59)	42 (31, 50)	45 (32, 53)	0.11
Diabetes duration (years)	20 (11, 29; 1–55)	18 (10, 29)	21 (12, 33)	0.20
Abdominal obesity^b^	29 (15)	21 (24)	8 (8)	0.002^c^
General obesity^d^	22 (12)	14 (16)	8 (8)	0.073^c^
HbA1c				
mmol/mol	63 (54, 71)	64 (54, 71)	62 (54, 68)	0.39
%	7.9 (7.1, 8.6)	8.0 (7.1, 8.7)	7.8 (7.1, 8.4)	
Total cholesterol (mmol/l)	4.6 (4.1, 5.1)	4.6 (4.1, 5.4)	4.6 (4.1, 5.1)	0.39
LDL-cholesterol (mmol/l)	2.8 (2.4, 3.3)	2.7 (2.4, 3.3)	2.8 (2.4, 3.3)	0.97
Triglycerides (mmol/l)	0.9 (0.6, 1.2)	0.8 (0.6, 1.3)	0.9 (0.7, 1.2)	0.83
HDL-cholesterol (mmol/l)	1.6 (1.3, 1.8)	1.6 (1.4, 1.9)	1.5 (1.3, 1.7)	0.040
Systolic BP (mm Hg)	120 (115, 130)	120 (110, 130)	125 (120, 130)	0.006
Diastolic BP (mm Hg)	70 (70, 75)	70 (65, 75)	70 (70, 80)	0.009
Hypoglycaemia (severe episodes)	9 (5)	5 (6)	4 (4)	0.73^c^
Smoking^e^	16 (9)	5 (6)	11 (11)	0.30^c^
Physical inactivity^f^	19 (10)	9 (11)	10 (10)	0.81^c^
Physical activity (times/week)				
> 5	63 (38)	32 (39)	37 (36)	0.74^g^
3–5	59 (32)	27 (33)	32 (32)	
1–2	36 (20)	14 (17)	22 (22)	
< 1	19 (10)	9 (11)	10 (10)	
Depression	20 (10)	8 (9)	12 (12)	0.64^c^
Alexithymia	30 (16)	17 (20)	13 (12)	0.23^c^
Antidepressants	13 (7)	9 (10)	4 (4)	0.087^c^
Continuous subcutaneous insulin infusion	19 (10)	11 (13)	8 (8)	0.33^c^
Lipid lowering drugs	91 (48)	39 (45)	52 (50)	0.56^c^
Antihypertensive drugs	59 (31)	21 (24)	38 (36)	0.084^c^
Cardiovascular complications	7 (4)	2 (2)	5 (5)	0.46^c^

Data are n (%) or median (q_1_, q_3_; min–max). ^a^Mann–Whitney *U* test unless otherwise indicated. ^b^WC (meters): women ≥ 0.88/men ≥ 1.02. ^c^Fisher’s exact test. ^d^BMI ≥ 30 kg/m^2^. Missing values (all/women/men): ^e^n = 5/3/2; ^f^n = 7/4/3. ^g^Linear-by-linear association

Comparisons of median MSC levels and comparisons of the distribution of high, intermediate and low MSC levels between women and men, and between four age categories are presented in Table 2. The prevalence of high MSC was 1.7 higher in the women than in the men (43% versus 26%), whereas the prevalence of both intermediate and low MSC were lower in the women (*p* = 0.048). In the women, there was no correlation between MSC levels and age categories (*p* = 0.75). In the men, there was a correlation between MSC levels and age categories (*p* = 0.027), and the prevalence of high MSC increased by each age category.

**Table 2:** Comparisons of MSC levels between men and women, and between four age categories presented for each sex

	All	Women	Men	*P* ^a^	Women				*P* ^b^	Men				*P* ^b^
Age (years)	18–59	18–59	19–59		18–< 30	30–< 40	40–< 50	50–59		19–< 30	30–< 40	40–< 50	50–59	
*N*	190	86	104		19	19	25	23		21	16	28	39	
MSC (nmol/l)	5.0 (3.1, 7.6)	5.6 (3.2, 8.2)	4.6 (3.1, 6.9)	0.072	6.5 (2.9, 7.9)	5.4 (3.2, 7.9)	5.3 (3.1–9.0)	5.8 (3.6, 9.4)	0.87	4.6 (2.8–5.3)	3.2 (2.5, 4.7)	5.2 (3.1, 9.4)	5.3 (3.6, 8.6)	0.049
High MSC^d^	64 (32.6)	37 (43.0)	27 (26.0)	0.048^c^	9 (47.4)	6 (31.6)	12 (48.0)	10 (43.5)	0.75 ^c^	2 (9.5)	3 (18.8)	9 (32.1)	13 (33.3)	0.027^c^
Inter–mediate MSC^e^	64 (33.7)	24 (27.9)	40 (38.5)		4 (21.1)	7 (36.8)	6 (24.0)	7 (30.4)		11 (52.4)	3 (18.8)	10 (35.7)	16 (41.0)	
Low MSC^f^	62 (33.7)	25 (29.1)	37 (35.6)		6 (31.6)	6 (31.6)	7 (28.0)	6 (26.1)		8 (38.1)	10 (62.4)	9 (32.1)	10 (25.6)	

Data are presented as median (q_1_, q_3_) midnight salivary cortisol (MSC) or N (%). ^a^Mann–Whitney *U* test unless otherwise indicated. ^b^Kruskal–Wallis H test unless otherwise indicated. ^c^Linear by linear association. ^d^≥ 6.7 nmol/l, ^e^≥ 3.7 to < 6.7 nmol/l, ^f^< 3.7 nmol/l

Comparisons between patients with and without abdominal obesity are presented for women and men in Table 3. The 21 women with abdominal obesity compared to the 65 non-obese women, had higher median MSC (*p* = 0.030). Comparisons also showed that the prevalence of high MSC was 1.7 higher in the abdominally obese (62% versus 37%), the prevalence of intermediate MSC was almost equal (33% versus 26%), and the prevalence of low MSC was 7.4 times lower in women with abdominal obesity (5% versus 37%) (*p* = 0.007). The prevalence of antidepressants was almost 6 times higher in the abdominally obese women compared to the non-obese women (29% versus 5%) (*p* = 0.006). Comparisons between the eight men with and the 96 men without abdominal obesity did not show any significant differences regarding MSC levels or use of antidepressants.

**Table 3:** Comparisons between patients with and without abdominal obesity presented for each sex

	Abdominal obesity					
	Women			Men		
	Yes	No	*p* ^a^	Yes	No	*p* ^a^
*N*	21	65		8	96	
Age (years)	45 (32, 50)	40 (31, 51)	0.81	52 (37, 58)	44 (32, 52)	0.17
Diabetes duration (years)	23 (13, 28)	18 (9, 29)	0.59	20 (10, 44)	21 (12, 33)	0.90
MSC^b^ (nmol/l)	7.1 (5.1, 8.8)	5.0 (2.9, 7.8)	0.030	3.8 (2.5, 5.4)	4.8 (3.1, 7.2)	0.37
MSC^b^						
High^c^	13 (62)	24 (37)	0.007^f^	1 (12)	26 (27)	0.29^f^
Intermediate^d^	7 (33)	17 (26)		3 (38)	37 (38)	
Low^e^	1 (5)	24 (37)		4 (50)	33 (34)	
Smoking^g^	1 (5)	4 (6)	> 0.99^h^	1 (12)	10 (11)	> 0.99^h^
Physical inactivity^i^	4 (19)	5 (8)	0.22^h^	0	10 (11)	> 0.99^h^
Physical activity^i^ (times per week)						
> 5	7 (33.3)	25 (41)	0.31^f^	2 (25)	35 (38)	0.82^f^
3–5	7 (33.3)	20 (33)		3 (37.5)	29 (31)	
1–2	3 (14.3)	11 (18)		3 (37.5)	19 (20)	
< 1	4 (19)	5 (8)		0	10 (11)	
Depression	2 (10)	6 (9)	> 0.99^h^	2 (25)	10 (10)	0.23^h^
Alexithymia	6 (29)	11 (17)	0.34^h^	3 (38)	10 (10)	0.060^h^
Antidepressants	6 (29)	3 (5)	0.006^h^	0	4 (4)	> 0.99^h^

Data are n (%) or median (q_1_, q_3_). ^a^Mann–Whitney *U* test unless otherwise indicated. ^b^Midnight salivary cortisol, ^c^≥ 6.7 nmol/l, ^d^≥ 3.7 to  < 6.7 nmol/l, ^e^< 3.7 nmol/l. ^f^Linear-by-linear association. Missing values (women/men): ^g^n = 3/2. ^h^ Fisher’s exact test. Missing values (women/men): ^i^n = 4/3

In women, median (q_1_, q_3_) WC (meters) was for low MSC: 0.76 (0.72, 080); for intermediate MSC: 0.79 (0.72, 0.89); and for high MSC: 0.81 (0.75, 0.94) (*p* = 0.028). In the men, the medians of WC did not differ between the three MSC levels (*p* = 0.48).

Associations with abdominal obesity are presented for all 190 patients in Table 4. Female sex (AOR 3.4 (CI 1.4–8.2)) and the use of antidepressants (AOR 4.3 (CI 1.2–14.8)) were associated with abdominal obesity. Associations with abdominal obesity are presented sex specified in Table 5. In the 86 women, high MSC (AOR 18.4 (CI 1.9–181)) and the use of antidepressants (AOR 12.2 (CI 2.0–73.6)) were associated with abdominal obesity (Table 5). In the 104 men, alexithymia (AOR 5.2 (CI 1.1–24.9)) was associated with abdominal obesity.

**Table 4:** Factors associated with abdominal obesity presented for all the 190 patients with T1D

	Abdominal obesity			
	COR (95% CI)	*p*	AOR (95% CI)	*p* ^a^
Gender (women)	3.9 (1.6–9.3)	0.002	3.4 (1.4–8.2)	0.008
Age (per year)	1.01 (0.98–1.04)	0.60	1.01 (0.97–1.05)	0.57
Diabetes duration (per year)	1.00 (0.97–1.04)	0.77	–	–
MSC^b^				
High	3.2 (1.1–9.5)	0.037	2.5 (0.8–7.9)	0.11
Intermediate	2.1 (0.7–6.6)	0.20	2.1 (0.6–6.9)	0.22
Low	1		1	
Hypoglycaemia	1.6 (0.3–8.3)	0.56	–	–
Smoking	0.8 (0.2–3.7)	0.76	–	–
Physical inactivity	1.5 (0.5–4.8)	0.51	–	–
Depression	1.4 (0.4–4.7)	0.54	–	–
Alexithymia	3.0 (1.2–7.5)	0.018	2.5 (0.9–6.5)	0.067
Antidepressants	5.7 (1.8–18.6)	0.004	4.3 (1.2–14.8)	0.023

N^a^ = 190; ^a^Multiple logistic regression analysis (Backward Wald): variables with *p*-values ≤ 0.10 for the CORs and age were included in the analyses; Nagelkerke R Square: 0.167; Hosmer and Lemeshow test: 0.488. ^b^Midnight salivary cortisol

**Table 5:** Factors associated with abdominal obesity presented separately for 86 women and 104 men with T1D

	Abdominal obesity							
	Women				Men			
	COR (95% CI)	*P*	AOR (95% CI)	*P* ^a^	COR (95% CI)	*P*	AOR (95% CI)	*P* ^b^
Age (per year)	1.00 (0.96–1.05)	0.87	0.99 (0.94–1.04)	0.63	1.05 (0.98–1.13)	0.20	1.05 (0.97–1.12)	0.22
Diabetes duration (per year)	1.01 (0.97–1.05)	0.67	–	–	1.01 (0.96–1.07)	0.62	–	–
MSC^c^								
High	13 (1.6–107)	0.017	18.4 (1.9–181)	0.013	0.7 (0.1–3.2)	0.62	–	–
Intermediate	9.9 (1.1–88)	0.040	10.3 (1.0–107)	0.051	0.3 (0.03–3.0)	0.32	–	–
Low	1		1		1		–	–
Hypoglycaemia	0.8 (0.1–7.2)	0.81	–	–	4.4 (0.4–48.3)	0.22	–	–
Smoking	0.8 (0.1–7.4))	0.83	–	–	1.2 (0.1–10.8)	0.87	–	–
Physical inactivity	2.6 (0.6–10.9)	0.18	–	–	–	> 0.99	–	–
Depression	1.0 (0.2–5.6)	0.97	–	–	2.9 (0.5–16.2)	0.23	–	–
Alexithymia	2.0 (0.6–6.2)	0.25	–	–	5.2 (1.1–24.9)	0.041	5.2 (1.1–24.9)	0.041
Antidepressants	8.3 (1.9–36.9)	0.006	12.2 (2.0–73.6)	0.006	–	> 0.99	–	–

N = ^a^86/^b^104; ^a,b^ Multiple logistic regression analysis (Backward Wald): variables with *p*-values ≤ 0.10 for the CORs and age were included in the analyses; Nagelkerke R Square: ^a^ 0.299/ ^b^0.082; Hosmer and Lemeshow test: ^a^0.639/^b^0.085. ^c^Midnight salivary cortisol

## Discussion

The main findings of this cross-sectional study were that female sex and the use of antidepressants were associated with abdominal obesity in all the patients with T1D, and that high MSC (≥ 6.7 nmol/l) and the use of antidepressants were independently associated with abdominal obesity in the women. The prevalence of abdominal obesity was three times higher in the women than in the men with T1D. In the men, only alexithymia was associated with abdominal obesity.

The first strength of this study was that the population of patients with T1D was well-defined. Patients with severe somatic or psychiatric comorbidities, pregnancy, and/or substance abuse were excluded. Of importance is that no patients who had been diagnosed with Cushing´s disease/syndrome were included. All patients using systemic corticosteroids, as well as patients where contamination with topical steroids were suspected were also excluded. Second, we have previously controlled that the MSC levels did not differ between people using and not using inhaled steroids [17]. Third, non-response analyses were previously performed, and no differences were found between those who delivered MSC samples and those who did not regarding age, diabetes duration, sex, abdominal obesity, serum lipids, blood pressure, smoking, physical inactivity, depression, or use of antidepressants [17]. Forth, we controlled for relevant variables such as smoking, physical inactivity, severe hypoglycaemia episodes, depression, alexithymia and age, which according to previous research are risk factors for abdominal obesity and/or contribute to increased cortisol secretion [16, 17, 25, 41]. We did not adjust for metabolic or cardiovascular variables that according to previous research are secondary to obesity [5, 6, 42]. Fifth, we checked that the sex differences with higher MSC and abdominal obesity in the women were not due to age. The women were not older than the men, and the distribution of high MSC did not differ between the four different age categories in the women. Only in the men, the prevalence of high MSC increased by each age category. Sixth, to our knowledge, there are no previous studies exploring links between MSC secretion, the use of antidepressants, sex differences and abdominal obesity in T1D patients. Seventh, we had access to both WC measurements and BMI. As WC measurements and the presence of abdominal obesity provide more information about risks for cardiometabolic disease than BMI [5, 43], we made the deliberate choice to include abdominal obesity instead of general obesity in the analyses.

The first limitation was that just 67% of the patients included in our research of abdominal obesity delivered MSC samples [28, 29]. Second, only one MSC sample was collected from each patient. Due to the inconvenience of midnight sampling, we anticipated a lower participation rate if we had demanded repeated samplings. Third, we did not perform any dexamethasone suppression tests, and Cushing´ syndrome can´t be excluded. A significantly higher rate of dexamethasone non-suppression has been demonstrated in people with diabetes [13]. Forth, quantification of physical activity was based on self-reported data. It is known that this parameter can be overestimated by the patients. Interviews were however done by skilled nurses and physicians who perform these interviews regularly. This method to assess levels of physical activity is used in the S-NDR, and has previously been proved to be valuable [30]. Fifth, we previously showed that physical inactivity was associated with abdominal obesity [28], but due to the limited number of participants, physical inactivity was no longer associated with abdominal obesity. Sixth, the number of men with abdominal obesity was low. Despite the low number, alexithymia was still associated with abdominal obesity in the men as in our previous research [28]. Nevertheless, potential associations between MSC, antidepressants and abdominal obesity ought to be further explored in a larger population of men with T1D. Seventh, the number of patients using antidepressants was low, with the consequence that subanalyses of use of antidepressants were not performed. Eighth, dietary habits were not included, but if they were, we would still not know whether the dietary habits were primary or secondary to the increased cortisol secretion in the abdominally obese women. Previous research has shown that glucocorticoids act to control food intake, in particular they are known to increase the consumption of foods enriched in fat and sugar [9]. Ninth, there were no data available regarding menopause from the participating women. But as there were no correlations between the cortisol levels and age categories in the women, there was no indication that menopause was of particular importance for determining the MSC levels.

As previously mentioned, there are several types of disturbances of the HPA axis observed in patients with T1D [8, 13–15]. The exaggerated adrenarche and altered cortisol metabolism observed in T1D children, may possibly result in persisting hyperandrogenic symptoms in women, including abdominal obesity [8]. We showed that female sex was associated with abdominal obesity in adults, which is in line with previous findings in children and adolescents with T1D [7]. Our results also support findings in previous research of obesity in non-diabetic populations, which showed that increased cortisol secretion is involved in the development of obesity, and that there are indications of a sex-dependent role of glucocorticoids in the pathophysiology of obesity [6, 9, 18]. Since this is a cross-sectional study, we cannot draw any conclusions whether the higher MSC levels are the cause of abdominal obesity. However, in previous research it has been demonstrated that HPA-axis activation with increased adrenal cortisol secretion can induce accumulation of fat cells and contribute to abdominal obesity [6, 9, 10, 18]. Nevertheless, once abdominal obesity is induced, there is evidence of increased conversion of inactive cortisone to active cortisol by the enzyme 11β-HSD1 in the adipocytes, further exacerbating obesity [6, 10]. Also at this level, sex differences have been demonstrated with upregulation of 11β-HSD1 by 17β-estradiol in women and by androgens in men [12]. Whether the adipocyte cortisol metabolism influences circulating cortisol levels is not clear. There are indications of a feedback loop between circulating cortisol and adipocyte cortisol metabolism, but further research is necessary to clarify the interactions between general cortisol activity and adipose tissue-specific expression of 11β-HSD1 [10].

Our research also supports the hypothesis that the use of antidepressants is a contributory factor to obesity [22]. However, we do not suggest that antidepressants should be avoided as antidepressants reduce mortality among patients with diabetes and depression [44].

The prevalence of obesity has increased dramatically since the introduction of intensified insulin therapy for people with T1D [7, 45]. All patients in this study had intensified insulin therapy administered either by MDII or by CSII. The prevalence of CSII did not differ between women and men, so our findings cannot be explained by different administration techniques. Neither did the prevalence of physical inactivity, smoking or severe episodes of hypoglycaemia differ between women and men.

There are several unanswered questions and indications for further research. The increased prevalence of high MSC secretion and of abdominal obesity which we found in the women with T1D might contribute to the increased risk for coronary artery calcification and cardiovascular mortality previously demonstrated in women with T1D [4, 46]. Therefore, we plan a 10-year follow up of cardiovascular complications for the patients included in this study. Further, if the increased cortisol secretion is due to environmental stress, what can be done to reduce the stress in the women? Whether antidepressants control food intake and energy expenditure, is another subject for further research. It would be of interest to explore which subclasses of antidepressants that show the best effects on depression, and the least impact on obesity and other metabolic variables in men and women with T1D. As abdominal obesity has been associated with increased testosterone levels in non-diabetic women [6], and as exaggerated adrenarche has been observed in children with T1D [8], it would be of interest to compare the testosterone levels between the T1D women with and without abdominal obesity. Finally, we suggest that MSC levels should be explored in patients with T1D and abdominal obesity in clinical practice. We also suggest, for both clinical and research reasons, that dexamethasone tests should be performed in patients with elevated MSC to exclude or confirm Cushing’s syndrome.

## Conclusions

Clear sex differences were demonstrated with a distinct higher prevalence of abdominal obesity, and a distinct higher prevalence of high midnight salivary cortisol in the women with type 1 diabetes. High midnight salivary cortisol secretion and the use of antidepressants were independently associated with abdominal obesity in the women with type 1 diabetes. Alexithymia was the only demonstrated risk factor for abdominal obesity in the men with type 1 diabetes. The increased prevalence of high midnight salivary cortisol secretion which was demonstrated in the women with abdominal obesity, might contribute to the excess risk of cardiovascular complications and death observed in women with type 1 diabetes.

## Data Availability

All data are saved at SPSS files for at least 10 years at the Department for Research and Development, Region Kronoberg, Växjö, Sweden. The data sets are not publicly available as individual privacy could be compromised. The data set is available from the corresponding author upon reasonable request.

## Abbreviations

AHD: antihypertensive drugs
AOR: adjusted odds ratio
COR: crude odds ratio
CSII: continuous subcutaneous insulin infusion
DBP: diastolic blood pressure
LLD: lipid lowering drugs
MDII: multiple daily insulin injections
MSC: midnight salivary cortisol
SBP: systolic blood pressure
WC: waist circumference
